# Urinary tract infections in culled sows from Greek herds: prevalence and associations between findings of histopathology, bacteriology and urinalysis

**DOI:** 10.1186/s40813-021-00212-3

**Published:** 2021-04-19

**Authors:** Mihaela Cernat, Vassilis Skampardonis, Georgios A. Papadopoulos, Fotios Kroustallas, Sofia Chalvatzi, Evanthia Petridou, Vassilios Psychas, Christina Marouda, Paschalis Fortomaris, Leonidas Leontides

**Affiliations:** 1 Department of Epidemiology, Biostatistics and Economics of Animal Production, School of Veterinary Medicine, University of Thessaly, 43132 Karditsa, Greece; 2 Laboratory of Animal Husbandry, Faculty of Veterinary Medicine, School of Health Sciences, Aristotle University of Thessaloniki, 54124 Thessaloniki, Greece; 3 Laboratory of Microbiology and Infectious Diseases, Faculty of Veterinary Medicine, School of Health Sciences, Aristotle University of Thessaloniki, 54124 Thessaloniki, Greece; 4 Laboratory of Pathology, Faculty of Veterinary Medicine, School of Health Sciences, Aristotle University of Thessaloniki, 54124 Thessaloniki, Greece

**Keywords:** Sow, Cystitis, Bacteriuria, Urinalysis, Proteinuria, Turbidity

## Abstract

**Background:**

Urinary tract infections (UTI) of sows characterized by cystitis, which may progress to ureteritis and pyelonephritis, can affect their productivity, longevity and welfare. In this study, we determined the prevalence of UTI by histopathology and bacteriology. Furthermore, we investigated possible associations between histologically confirmed cystitis and the results of urinalysis and urine cultures in culled sows from three farrow-to-finish herds in Greece.

**Materials and methods:**

One hundred eighty-five routinely culled sows were included in the study. Their urinary bladder was collected from abattoirs and subjected to histopathology. Furthermore, urinalysis and urine cultures were performed on urine samples aseptically collected from the bladders.

**Results:**

Histologically confirmed cystitis was evident in 85/185 (45.94%) culled sows. Among those, 44 (51.76%) suffered from acute and 41 (48.24%) from chronic inflammation. The majority of positive urine cultures were due to colonization of the urinary tract with *E.coli*, which accounted for 55.81% of the total cases, followed by *Staphylococcus* spp. which accounted for 18.60% of detected infections. Evidence of cystitis was associated with bacteriuria and sows with bacteriuria were 2.30 (*P* = 0.03, 95% CI: 1.10–4.83) times more likely to have histologically confirmed cystitis compared to sows with negative urine cultures. Bacteriuria was associated with proteinuria (*P* < 0.01, OR = 9.72, 2.63–35.88), increased urine pH (*P* < 0.01, 3.40, 1.10–10.56) and presence of sediment (*P* < 0.01, 6.00, 1.50–23.76) in urine. Sows with proteinuria had 9.72 (*P* < 0.01, 2.63–35.88) times higher odds of bacteriuria than those without. Histologically defined cystitis was associated with proteinuria (*P* < 0.01, 2.03–13.20) and decreased urine pH (*P* < 0.01, 0.13–0.72). Sows with proteinuria were 5.18 (*P* < 0.01, 2.03–13.2) times more likely to have histological lesions consistent with cystitis, than those without. For one unit increase in pH, it was 3.20 (*P* = 0.006, 1.39–7.69) times less likely for a sow to have chronic or acute cystitis compared to absence of cystitis.

**Conclusion:**

In the studied population, UTI affected almost one out of two culled sows. Bacteriuria, which was more common among sows with UTI than those without, was mainly ascribed to members of the intestinal and environmental bacteria. Proteinuria and the existence of urine sediment which were associated with UTI, could be used as proxy traits for UTI in live sows**.**

## Introduction

Urinary tract infections (UTI) of sows, characterized by cystitis (inflammation of the urinary bladder) that may progress to ureteritis (inflammation of the ureters) and pyelonephritis (inflammation of the renal pelvis), are major health and welfare issues with economic impact to the swine industry because of sudden death, partly attributed to pyelonephritis and septicemia [1], or sub-optimal reproductive performance [2–8]. UTI are associated with colonization of the normally sterile urinary tract with bacteria, which are most usually members of the fecal microbiota, ascending from the vagina and distal urethra [9], mainly prepartum [10]. In sows with UTI, ureteric valves are shortened from normal length. In case of cystitis, bacteria can reflux back to the kidney [11]. Bacteriuria (culture positive urine), however, may not be always detected in samples from sows with UTI or may be detected from sows without UTI [7, 12].

The prevalence of UTI which was estimated in live sows [13–16] as well as at abattoirs [3, 12, 17], by either reagent strip tests, urine culture or histopathology, vary considerably from 15.8 [15] to 58% [16]. There are many reasonable explanations for this wide variation in estimates which challenge the comparability among study results; the most important being the different case definitions, and the different parity structure of the studied sow populations. The reagent strip tests which have been applied in some of the on-farm studies are ineffective, especially in the diagnosis of chronic UTI [12], thus underestimating UTI prevalence. Madec and Leon (1992) detected UTI in 23.5% of sows of parity ≥5 whereas none of first-parity sows had evidence of UTI. Earlier studies [18–21], investigating the causes of sow mortality, reported the frequency of UTI, after defining diagnosis either based on gross pathology or on bacterial isolation, ranging from approximately 6 [20, 21] to 19.4% [18]. Nonetheless, UTI remain largely under-diagnosed and therefore untreated [7, 22], despite being associated with several periparturient clinical problems which may lead to producers’ decision for early culling [3–6] of sows. The most common bacteria associated with cases of UTI were *E. coli*, *Streptococci*, *Staphylococci*, and *Actinobaculum suis*, either alone or in combination [23]. In diagnosed cases of UTI, clinical management included treatment with broad spectrum antibiotics [11].

Diagnosis of UTI in live sows is based on urinalysis and urine culture results. Histopathology, being the “gold standard” for diagnosis, is the method of choice in dead animals. Compared to the “gold standard”, urinalysis and bacteriology demonstrated low sensitivity but relatively high specificity in detecting UTI [12]. Because of increased possibilities of bacterial contamination during sampling in live animals, the positive predictive value of bacteriological culture was reported as being relatively low [3].

To the best of the authors’ knowledge, the prevalence of UTI has not been reported in multiparous sows of Greek herds. Similarly, the bacteria most frequently involved and their antibiotic sensitivity profile were not determined before. The latter is important, considering anecdotal reports highlighting the significance of UTI for sow’s longevity and productivity, especially during the warmest period of the year from early May to late September. Thus, the objective of the present study was threefold: i) to estimate the prevalence of histopathologically confirmed cystitis in culled sows from three Greek farrow-to-finish herds, ii) to perform urinalysis and urine culture on samples from culled sows of the herds, isolate the bacteria involved in bacteriuria incidents and determine their sensitivity profile, and iii) to investigate possible associations between cystitis and the results of urinalysis and urine culture.

## Materials and methods

### Study population

Three indoor, farrow-to-finish herds were involved in our study that lasted from January 2019 until April 2020. The owners gave their written consent for participation in the study. All herds complied with EU directive 2001/88/DC on animal welfare. The herds comprised 250 (Herd A), 350 (Herd B) and 370 sows (Herd C), with PIC, Danbred and Topigs Norvsin genetic lines, respectively. Culling of sows was decided by the manager/owner of each herd without any intervention of investigators who were only informed about scheduled culling in order to organize sample collection from abattoirs.

### Animals and abattoir sampling protocol

Council Directive 93/119/EC on the protection of animals during slaughter was standard practice for all sows included in the study.

Almost 50% of sows routinely culled in the designated herds during the study period were included. Sample collection was performed at the abattoirs with minimum interference in the normal slaughtering process. Sow carcasses did not pass through scalding water; they were skinned immediately after stunning and exsanguination. Urinary bladders were removed before evisceration to minimize possible contamination from gut content. Immediately after removal, the neck of the bladder was fastened [12]. Subsequently, the surface of the bladder was surgically scrubbed and mid-flow urine was collected after gently shaking the bladder. Urine samples from each sow were collected in two sterile containers, one destined for urinalysis and the other for culture, from a small incision (approximately 1 cm) made in the fundus of the bladder with surgical blade. Urine samples and bladders were cooled immediately using portable thermo-insulated containers with ice packs and transferred to the laboratory for analysis. Macroscopic and microscopic evaluation was performed on all urine samples that arrived at the laboratory in acceptable condition within 6 to 12 h after collection.

### Macroscopic examination of bladders

**Fig. 1:**
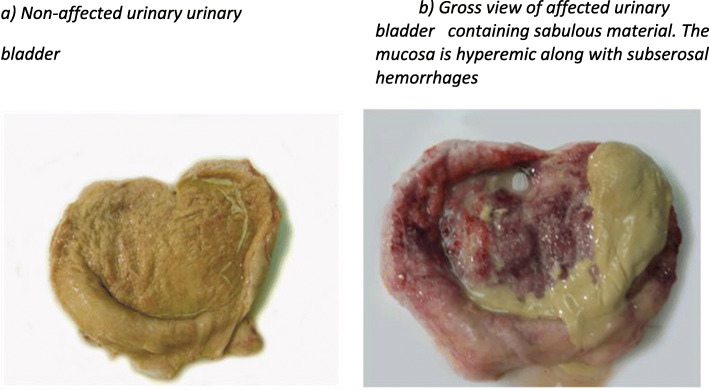
Macroscopic view of non-affected (**a**) and affected (**b**) urinary bladders, **a**) Non-affected urinary urinary bladder**. b**) Gross view of affected urinary bladder containing sabulous material. The mucosa is hyperemic along with subserosal hemorrhages

Urinary bladders were macroscopically examined, after dissection with scissors along their sagittal axis, from the neck to apex to expose the mucosa. Lesions recorded during gross examination of urothelium were: 1) superficial mucosal hyperemia and hemorrhages, 2) diffusely thickened, hyperplastic bladder wall and 3) lesions regarding the integrity of the urothelium, such as ulcerations, mucosal edema and fibrinopurulent exudate. Presence of concretions or calculi was recorded and subsequently examined microscopically. Thereafter, the bladders were thoroughly cleaned, and lesions were closely re-observed. Finally, they were categorized as either non-affected when they had no lesions or calculi, or as affected when lesions or concretions were observed (Fig. 1).

### Histopathological examination of bladders

**Fig. 2:**
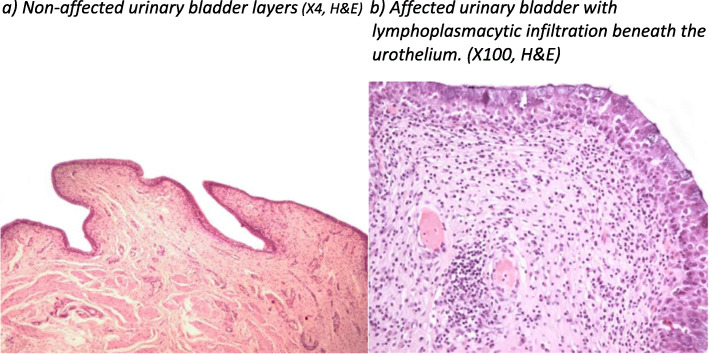
Microscopic view of non-affected (**a**) and affected (**b**) urinary bladder (H&E stain). **a**) Non-affected urinary bladder layers (X4, H&E) **b**) Affected urinary bladder with lymphoplasmacytic infiltration beneath the urothelium. (X100, H&E)

**Fig. 3:**
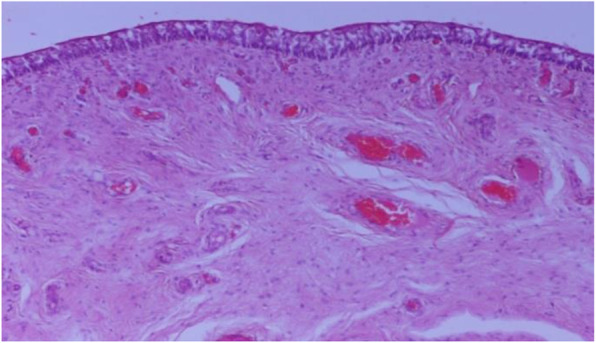
Urinary bladder with dilated and congested capillaries. Absence of inflammation. H&E stain × 100)

Full thickness representative samples from the neck and the fundus of all urinary bladders were obtained and fixed in 10% neutral-buffered formalin for minimum 48 h. Samples were further processed routinely, embedded in paraffin and sectioned at 4 μm thickness. Sections were stained with hematoxylin and eosin (H&E) and were assessed blindly by two pathologists. Distribution of lesions was defined as focal, multifocal or diffuse [12]. Cystitis was diagnosed when at least one focal area of inflammatory infiltration was observed. Urinary bladders without inflammatory infiltration were considered histologically normal (Fig. 2). Bladders with slaughter-related hyperemia were categorized as non-affected when hyperemia was not accompanied with additional lesions [24] (Fig. 3).

**Fig. 4:**
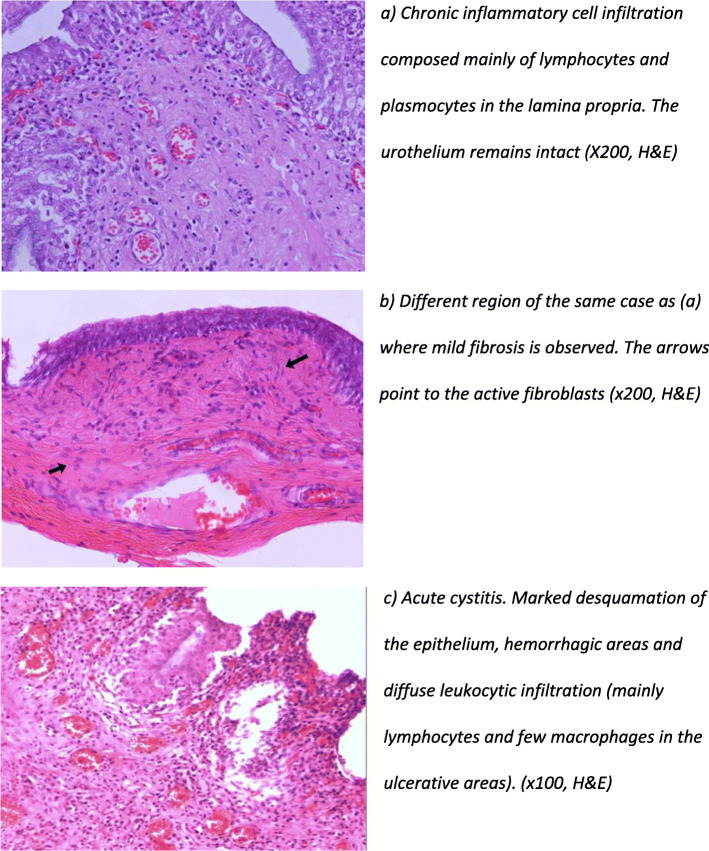
Chronic (**a,b**) versus acute (**c**) cystitis (hematoxylin and eosin stain). **a** Chronic inflammatory cell infiltration composed mainly of lymphocytes and plasmocytes in the lamina propria. The urothelium remains intact (X200, H&E). **b** Different region of the same case as (**a**) where mild fibrosis is observed. The arrows point to the active fibroblasts (× 200, H&E). **c** Acute cystitis. Marked desquamation of the epithelium, hemorrhagic areas and diffuse leukocytic infiltration (mainly lymphocytes and few macrophages in the ulcerative areas). (× 100, H&E)

Distinction between acute and chronic cystitis was determined by the type of cell infiltration (neutrophils and macrophages in acute and lymphocytes or plasma cells in chronic), the state of urothelium (necrosis and desquamation of the epithelium indicated an acute inflammation) and the degree of fibrosis in chronic conditions [25]. Cystitis was categorized as either acute when areas with necrosis and exfoliation of urothelial cells were observed along with mild or moderate superficial hyperemia and marked leukocytic mucosal infiltration, or chronic, when lymphoplasmacytic infiltration beneath the epithelium and areas with mild submucosal fibrosis were observed (Fig. 4).

Inflammation severity was categorized according to distribution, intensity/degree of leukocytic lamina propria inflammation and infiltrated layers of bladder wall. In particular, inflammation was classified as: i) mild when focal inflammatory infiltration was observed, but did not extend to deeper layers of the wall bladder, ii) moderate when bladders had multifocal or diffuse distribution of inflammatory infiltration and prominent leukocytic infiltration of the lamina propria, and iii) severe when diffuse distribution of inflammation, marked infiltration of mononuclear cells, and exfoliation of the epithelium, with or without mucosal hemorrhages, was noticed [12, 24, 25].

### Urinalysis

For consistency and comparability of results among samples, especially for the examination of urine sediment’s characteristics and constituents, we collected 10 ml of urine from all sampled bladders [26, 27]. Initially, the urine samples were transferred into conical tip tubes and were evaluated for turbidity. Urine pH was measured by an electric pH meter (HD2105.1® delta OHM), and protein estimation was done with the nitric acid method [28]. Then, samples were centrifuged at 1500 rpm for 5 min and subsequently, the presence or absence of sediment was recorded. The infranatant product of centrifugation was observed under light microscope (× 40 and × 100). Cellular components (red blood cells and leukocytes) were recorded [12]. Crystals were identified and characterized based on their shape, size and consistency, using high power magnification (× 200 and × 400) and infrared spectroscopy [29, 30].

### Urine culture

Samples for culture were transferred into 1 ml conical tip tubes using a disposable pipette, after gently inverting the original container in order to mix the content. Subsequently, using a sterile loop or a capillary pipette, samples were inoculated onto Columbia agar containing 5% sheep blood, and onto MacConkey agar plates, and incubated at 37^o^ C for 24 h. Two separate samples were inoculated and incubated respectively in CO_2_ enriched atmosphere, for 24–48 h at 37 °C, and anaerobically for 48–96 h. The bacterial count per ml was assayed after spreading a standard quantity of urine onto 5% sheep blood agar, incubated aerobically for 24 h at 37 °C [31]. Pure or mixed cultures with more than 10^5 ^CFU/ml bacteria were considered positive [12]. Bacteria identification was performed using biochemical testing; their sensitivity to commonly prescribed antibiotics, namely ampicillin, amoxicillin + clavulanic acid, enrofloxacin, tetracycline, cephalexin, colistin, ceftazidime and erythromycin, was tested. Choice of antibiotics was based on routinely employed methodology of certified laboratories providing services to veterinary practitioners. Those antibiotics are commonly prescribed in practice in Greece.

#### Statistical analysis

All statistical analyses were performed using Stata 13.1 (Stata Statistical Software, College Station, Texas). Descriptive statistics of collected data were calculated. Kruskal-Wallis ANOVA was used to compare parity distributions among herds. Pearson’s *χ*^2^ tests were used to compare the proportion of sows with histologically confirmed cystitis and bacteriuria, among the three herds, across parities and among season of culling. One-way ANOVA was used to compare the average urine pH values across herds, parities and season of culling. A linear regression model was employed to investigate the possible association between the pH and the presence of crystals in urine. Pearson’s *χ*^*2*^ tests were additionally used to test for possible associations between urinalysis parameters and to compare the frequencies of antibiotic sensitivity of each isolated bacteria species among herds.

### Associations between histologically confirmed cystitis and urinalysis parameters

We employed an ordered logistic regression model to test any possible association between cystitis, classified as absent, acute or chronic, and urinalysis parameters. Cystitis, was the dependent variable, while urine turbidity, pH, presence of sediment, crystals, red blood cells, leukocytes and protein were used as independent covariates. For model building we followed the procedure described by Lo Fo Wang et al. (2004) [32]. All independent variables were initially screened one by one. During this process, a significance level of 0.25 was used [33]. Then variables with *P* < 0.25 were subjected simultaneously to a full model, subsequently reduced by backwards elimination [34] until only significant (*P* < 0.05) variables remained. Two-factor interactions were created between the remaining variables and subjected one at a time to the model. Finally, we tested previously deleted variables one-by-one to the final model, to ensure that no variable with significant impact to the model was omitted. The proportional odds assumption of ordered logistic regression models was tested using the *oparallel* post-estimation command [35]. Goodness of fit of the final model was tested with the *ologitgof* post-estimation command [36].

### Association between macroscopical lesions in urinary bladders and histologically confirmed cystitis

The evaluation of the association between macroscopical lesions on the urothelium of the bladders and histological lesions consistent with cystitis was evaluated in a logistic regression model. The presence or absence of histologically confirmed cystitis was the dependent variable while the presence or absence of gross lesions in the urinary bladder was the independent variable.

### Associations between bacteriuria and urinalysis parameters

For the investigation of possible associations between bacteriuria and the parameters examined in urinalysis we employed logistic regression modelling. Model building followed the procedure previously described.

### Association between bacteriuria and histologically confirmed cystitis

The significance of the association between bacteriuria and the presence of cystitis was tested in a logistic model. In addition, an ordinal logistic regression model was employed to investigate any association of bacteriuria with inflammatory status of the bladder (absent, acute or chronic cystitis). Model building followed the procedure described above.

## Results

A total of 185 culled sows were sampled. Fifty-seven (57) originated from herd A, sixty-four (64) from herd B and sixty-four (64) from herd C. Their parities ranged from 1 to 9, with a median of 6. Specifically, the median parity was 5 (range 1–7), 6 (range 1–9) and 6 (range 1–8) in sows from herd A, B and C, respectively; parity distributions did not differ (*P* = 0.33) among herds.

Gross examination of urinary bladders revealed that 136/185 (73.50%) had no remarkable lesions, while in the remaining, congestion was observed in 39/185 (21.08%), hemorrhages were noted in 4/185 (2.16%), concretions were present in 4/185 (2.16%), while 2/185 (1.08%) urinary bladders had simultaneously hemorrhages, congestion and concretions. Histologically, cystitis was evident in 85/185 (45.94%) sows. Among those, 44 (51.76%) suffered from acute and 41 (48.24%) from chronic inflammation. Among sows with chronic cystitis, the inflammation was mild (39/41, 95.12%) or moderate (2/41, 4.88%), while among cases of acute cystitis, 27/44 (61.36%) were classified as mild, 13/44 (29.55%) as moderate and 4/44 (9.09%) as severe. There were no significant differences in the prevalence of cystitis in sows among herds (*P* = 0.59), across different parities (*P* = 0.90) and among seasons of the year (*P* = 0.09).

Urinalysis data were available from 134 of the 185 sampled sows, because of the absence or inadequate quantity of urine in the bladders of the remaining 51 sows (Table 1). Turbid urine was found in 35/134 (26.12%) sows. Sediment was recorded in 53/134 (39.55%) sows. Leukocytes and red blood cells were identified in 20/134 (14.93%) and 19/134 (14.18%) sows, respectively; the urine of 11/134 (8.21%) sows had leukocytes and red blood cells. Crystals were detected in 46/134 (34.33%) sows. The main component of all but one of the urinary crystals identified, was calcium oxalate and the remaining was struvite.

**Table 1:** Results of urinalysis on samples collected from the urinary bladders of 134 culled sows from three farrow-to-finish Greek herds

Laboratory findings	In total (***n*** = 134)	Herd A (***n*** = 27)	Herd B (***n*** = 54)	Herd C (***n*** = 53)
**Urine appearance**				
Clear	99 (73.88%)	18 (66.67%)	41 (75.93%)	40 (75.47%)
Turbid	35 (26.12%)	9 (33.33%)	13 (24.07%)	13 (24.53%)
**Proteinuria**				
No	104 (77.61%)	20 (74.07%)	43 (79.63%)	41 (77.36%)
Yes	30 (22.39%)	7 (25.93%)	11 (20.37%)	12 (22.64%)
**Sediment**				
Absence	81 (60.45%)	13 (48.15%)	32 (59.26%)	36 (67.92%)
Presence	53 (39.55%)	14 (51.85%)	22 (40.74%)	17 (32.08%)
**Leucocytes**				
Absence	114 (85.07%)	24 (88.89%)	46 (85.19%)	44 (83.02%)
Presence	20 (14.93%)	3 (11.11%)	8 (14.81%)	9 (16.98%)
**Red blood cells**				
Absence	114 (85.07%)	24 (88.89%)	43 (79.63%)	47 (88.68%)
Presence	20 (14.93%)	3 (11.11%)	11 (20.37%)	6 (11.32%)
**Crystals**				
Absence	88 (65.67%)	16 (59.26%)	35 (64.81%)	37 (69.81%)
Presence	46 (34.33%)	11 (40.74%)	19 (35.19%)	16 (30.19%)
**Average pH (±SD)**	7.27 (± 0.043)	7.33 (± 0.111)	7.23(±0.620)	7.30 (± 0.071)

The average urine pH did not differ either among herds (*P* = 0.37), or across parities (*P* = 0.86) or among season of culling (*P* = 0.42). The presence of crystals was associated with urine pH (*P* < 0.001); sows with crystals in their bladder had higher pH values by on average 0.32 (*P* < 0.001, 0.16–0.49) units. Urine turbidity was associated with presence of sediment (*P* < 0.001). Specifically, sows with turbid urine had 8.60 (95% CI: 3.60–20.49) times higher odds of having urine sediment compared to sows with clear urine.

Urine-culture data were available from 171 sows. Overall, the frequency of positive urine cultures was 38/171 (22.22%) and it did not differ either among herds (*P* = 0.44), or across parities (*P* = 0.82) or among seasons of culling (*P* = 0.12). Cystitis was histologically confirmed in 23/38 (60.53%) of sows with bacteriuria.

The frequency of isolated bacteria species is presented in Table 2. Among positive urine samples there were 5/38 (13.16%) with mixed infections, whereas the remaining 33/38 (86.84%) were pure cultures. The sensitivity profile of isolated bacteria is shown in Table 3. The sensitivity patterns of the isolated bacteria did not differ among herds (the lowest *P* = 0.13).

**Table 2:** Frequency of isolated bacteria species in 38 culled sows with positive urine cultures. Mixed infections were observed in 5 sows

	In total	Herd A	Herd B	Herd C
***Escherichia coli***	24/43 (55.81%)	7/12 (58.33%)	12/19 (63.16%)	5/12 (41.67%)
***Enterococcus*** **spp.**	4/43 (9.30%)	2/12 (16.67%)	1/19 (5.26%)	1/12 (8.33%)
***Enterobacter*** **spp.**	4/43 (9.30%)	1/12 (8.33%)	2/19 (10.53%)	1/12 (8.33%)
***Staphylococcus*** **spp*.***	8/43 (18.60%)	2/12 (16.67%)	2/19 (10.53%)	4/12 (33.34%)
***Klebsiella*** **spp.**	1/43 (2.33%)	0/12 (0.00%)	1/19 (5.26%)	0/12 (0.00%)
***Actinobaculum suis***	2/43 (4.66%)	0/12 (0.00%)	1/19 (5.26)	1/12 (8.33%)

**Table 3:** Sensitivity of isolated bacteria to ampicillin (AMP), amoxicillin + clavulanic acid (AMC), enrofloxacin (ENR), tetracycline (TET), cephalexin (CN), colistin (COL), ceftazidime (CAZ) and erythromycin (ERY)

		% percentage of sensitivity							
Isolated bacteria	Total cases isolated	AMP	AMC	ENR	TET	CN	COL	CAZ	ERY
***Escherichia coli***	24	25	66.66	95.83	41.62	79.16	75	91.66	66.66
***Enterococcus*** **spp.**	4	0	75	100	25	75	100	100	100
***Enterobacter*** **spp.**	4	50	75	100	75	75	100	100	100
***Staphylococcus*** **spp*.***	8	25	62.5	87.5	25	87.5	100	87.5	62.5
***Klebsiella*** **spp.**	1	0	0	0	0	100	100	100	0
***Actinobaculum suis***	2	0	100	100	50	100	100	100	100

### Association between histologically confirmed cystitis and urinalysis parameters

After the screening process, five parameters, namely, urine turbidity, proteinuria, pH, presence of sediment and presence of leukocytes were selected for the full model. After model building, proteinuria and pH were significant. Their interaction was not statistically significant (*P* = 0.63). The assumption of proportionality of odds was not violated (*P* = 0.15, Brant test). Hosmer-Lemeshow (HL) (*P* = 0.28) and Pulkstenis-Robinson (PR) (*P* = 1.00) tests suggested an overall adequate fit of the model to the data. The odds of acute and chronic cystitis combined, versus absence of cystitis were 5.18 (*P* = 0.001, 95% CI: 2.03–13.20) times greater in the presence of proteinuria. Likewise, for one unit increase in pH, it was 3.20 (*P* = 0.006, 1.39–7.69) times less likely for a sow to have chronic or acute cystitis compared to absence of cystitis.

### Association between macroscopic lesions in urinary bladders and histologically confirmed cystitis

Urinary bladders with macroscopic lesions were 5.60 (*P* < 0.001, 2.70–11.80) times more likely to have histological lesions of cystitis, either acute or chronic, compared to those without gross lesions.

### Association between bacteriuria and urinalysis parameters

In the screening process, all urinalysis parameters were eligible for inclusion in the full model. After model building, proteinuria, presence of sediment and pH were retained. The 2-way interactions examined were not statistically significant. HL (*P* = 0.79) test suggested an overall adequate fit of the model to the data. Sows with proteinuria had 9.72 (*P* < 0.01; 2.63–35.88) times higher odds of bacteriuria than those without. Sows with sediment in their bladder had 6.00 (*P* = 0.01; 1.50–23.76) times higher odds of bacteriuria, compared to sows without sediment. Lastly, for one unit increase of urine pH it was 3.40 (*P* = 0.01; 1.10–10.56) times more likely for a sow to have bacteriuria.

### Association between bacteriuria and histologically confirmed cystitis

Sows with bacteriuria were 2.30 (*P* = 0.03; 1.10–4.83) times more likely to have histological lesions consistent with cystitis in their urinary bladders, than sows with negative urine cultures. Moreover, an association of bacteriuria with the inflammatory status of the bladder was detected; acute cystitis was 3.55 (*P* = 0.004; 1.48–8.50) times more likely to be accompanied by bacteriuria when compared to absence of cystitis. In contrast, the odds of bacteriuria did not differ (*P* = 0.39) either between sows with chronic or no cystitis or between sows with acute or chronic cystitis (*P* = 0.09).

## Discussion

The effect of diseases of the urogenital track, such as endometritis and cystitis, is considered an important determinant of farms’ economic output and animals’ welfare status [37]. Biksi et al. (2002) reported positive association between cystitis and endometritis, while Gmeiner et al. (2007) [38] suggested the existence of a bidirectional reservoir of infections, with non-specific and opportunistic pathogens, between the uterus and the urinary tract. In our study, we estimated the prevalence of UTI in culled sows from 3 swine herds in Greece through histopathological evaluation of the urinary bladder, urine culture and urinalysis. Furthermore, we investigated possible associations between histopathologically defined cystitis [3, 12] and the results of urinalysis and urine cultures.

The estimated prevalence of cystitis was within the range of previously studies in culled sows [3, 12, 17]. Similarly to findings of Bellino et al. (2013) and Grattarola et al. (2010), we found that bacteriuria was less frequent than histologically confirmed cystitis by almost 50%. In line with previous studies [16, 39] we detected *E.coli* as the leading cause of bacteriuria, accounting for approximately 50% of all cases, followed by *Staphylococcus* spp.; these bacteria were cumulatively responsible for over 70% of cases of bacteriuria. Most likely, these cases were the result of ascending infections which are considered common because of the female’s short urethra and the physiological relaxation of the sphincter muscle peripartum. These bacteria which likely originate from sows’ intestinal tract, contaminate the environment of pig houses and ascend and colonize the lower part of the urinary tract [12, 40, 41]. Interestingly, we found only 2 sows infected with *Actinobaculum suis*, a specific contaminant of the urinary tract with descending significance compared to data reported in the past mainly due the wide application of artificial insemination [12, 42].

*E. coli* and other Gram-negative (*Enterobacter* spp*.*) and -positive (*Staphylococcus* spp*., Enterococcus* spp*.*) isolates were not sensitive to most of the antibiotics tested. Although the range of antibiotics included in our sensitivity evaluation panel may be considered narrower than those of previous reports [16, 43], our results may indicate that there is a significant reduction of efficacy of commonly prescribed antibiotics in incidents of UTI in sows in Greek herds.

The results showed that almost 1 out of 3 tested sows had turbid urine. Among sows with bacteriuria, urine turbidity was recorded in almost 2 out of 3 sows. Urine turbidity was associated with presence of sediment, which in turn, was associated with crystalluria. Urine may sometimes contain less sediment compared to the amount observed in the bladder through ultrasonographic depiction [44]. However, our collection method of mid-stream urine after gentle shaking of urinary bladders for about a minute led to a rather homogenous distribution of urine sediment. We microscopically examined the detected sediment, and recorded all of its constituents; i.e., red blood cells, leukocytes and crystals. Crystals in urine may be either the result of abnormalities in the mineral composition of the feed, such as imbalance in calcium and phosphorus intake [45], and/or the result of insufficient water consumption and/or, alternatively, be induced by local infections [46].

The notion is that higher parity predisposes both to endometritis and to cystitis [6, 47–49]. In our study, parity was not associated either with histologically confirmed cystitis or with bacteriuria, similarly to reports from Biksi et al. (2002) [50] and Piassa et al. (2015) [16]. The debate among studies on the existence of an association between UTI and parity distribution of sows may be a reflection of differences in parity distribution of studied sow populations. We did not find any significant variation in cystitis or bacteriuria frequency among herds or among-season. Sows with proteinuria were more likely to have histological lesions consistent with acute or chronic cystitis than those without proteinuria; they were more likely to have positive urine cultures than those without proteinuria. Proteinuria, the presence of abnormal protein level in urine, is a consequence of either abnormal transglomerular passage of proteins, because of increased permeability of glomerular capillary wall and their subsequent impaired reabsorption by the epithelial cells of the proximal tubuli [51], or the consequence of urea breakdown by bacteria in an alkaline environment (urine pH > 8). Grahofer et al. (2020) in their recent report of biomarkers for diagnosis of cystitis in sows, identified and proposed the presence of proteinuria as one of the most reliable and sensitive urinalysis biomarkers, in comparison to the gold standard of histopathology. In human medicine, Carter et al. (2006) [52] also reported an association between proteinuria and bacteriuria but the causative relationship remained undefined. In swine, proteinuria was associated with renal damage in an experimental study of Ravnskov et al. (1975) [53]. Carr et al. (1995) reported that in cases of failure of lower urinary tract defenses, such as in the case of cystitis, bacterial disease processes could result in damage of ureterovesicular junction, leading to pyelonephritis, due to vesicoureteric reflux. Ureteric valves in UTI affected sows are shortened from their normal length, and bacteria from the cyst can reflux back to the kidney [11]. In this study we did not investigate the impact of potential co-existence of glomerulonephritis and cystitis but, given the detected association between proteinuria and increased risk of cystitis, future studies should attempt to investigate its clinical importance.

Although in previous studies [16, 41] researchers reported no correlation between presence of cystitis and urine pH, we found that higher pH values were associated with increased odds of bacteriuria or, with decreased odds for a sow to have lesions of acute and/or chronic cystitis, compared to having a normal bladder. It has been shown that alkaline pH supported the development of certain urinary sediment and was associated with bacteriuria. Nonetheless, a causative relationship between alkaline urinary pH and bacteriuria is difficult to establish because it is affected by several factors [23]. In some studies, the acid-base balance of the diet was correlated with urinary pH and total bacteria colony forming units in urine [48, 54]. Lowering of urine pH was proposed as a mean to inhibit or control urinary bacterial overgrowth [42]. In sows with urinary tract infection, urine alkalization may result from urea transformation into ammonia by the bacterial flora [54]. Therefore, high urine pH may be the result of the presence and the metabolism of bacteria, or may precede or even trigger the colonization of the urinary tract with bacteria. In the cross-sectional context of this study inferences about the exact role and the potential causal effect of urine pH in UTI are not justified.

Presence of sediment was associated with higher odds of bacteriuria. Kauffold et al. (2010), using ultrasonography, reported that sows with UTI, defined on the basis of high bacterial count and macroscopic/biochemical urine abnormalities, were more likely to have high or moderate amounts of sediment in urine than those without UTI. Recently, Grahofer et al. (2020), also concluded that evaluation of presence of sediment is useful to detect sows with cystitis and bacteriuria.

## Conclusion

In conclusion, in the present study we found that cystitis was quite frequent, affecting almost half of sows studied. The majority of sows with bacteriuria had histologically confirmed cystitis. We found that in most cases bacteria isolated from urine were mainly part of the normal intestinal flora which points to environmental and managerial involvement in the causality of UTI. Proteinuria was associated with both cystitis and bacteriuria, while urine turbidity was highly associated with bacteriuria; almost 2 out of 3 sows with bacteriuria had turbid urine. Therefore, urine turbidity and proteinuria appear as valuable diagnostic tools for clinical diagnosis of cystitis in sows.

## Data Availability

Available from senior author upon request.

## Abbreviations

UTI: Urinary tract infection
CI: Confidence interval
OR: Odds ratio
H&E: Hematoxylin and eosin
HL: Hosmer-Lemeshow
PR: Pulkstenis-Robinson
